# Hand Gesture Recognition in Automotive Human–Machine Interaction Using Depth Cameras

**DOI:** 10.3390/s19010059

**Published:** 2018-12-24

**Authors:** Nico Zengeler, Thomas Kopinski, Uwe Handmann

**Affiliations:** 1 Hochschule Ruhr West, University of Applied Sciences, 46236 Bottrop, Germany; 2 South Westphalia University of Applied Sciences, 59872 Meschede, Germany

**Keywords:** neural networks, hand gesture recognition, time-of-flight sensors, automotive human–machine interaction

## Abstract

In this review, we describe current Machine Learning approaches to hand gesture recognition with depth data from time-of-flight sensors. In particular, we summarise the achievements on a line of research at the Computational Neuroscience laboratory at the Ruhr West University of Applied Sciences. Relating our results to the work of others in this field, we confirm that Convolutional Neural Networks and Long Short-Term Memory yield most reliable results. We investigated several sensor data fusion techniques in a deep learning framework and performed user studies to evaluate our system in practice. During our course of research, we gathered and published our data in a novel benchmark dataset (REHAP), containing over a million unique three-dimensional hand posture samples.

## 1. Introduction

Humans may communicate nonverbally with hand movements carrying certain symbolic meanings, a behaviour we want to abstract, formalise and use for driver–vehicle interaction. Several hand sign languages exist and usually feature sets’ context-sensitive hand gestures. In order to ground hand sign symbolism, we may use visual information about hand postures as well as depth data obtained by adequate Time-of-Flight (ToF) sensors like the Camboard Nano sensors we used. Uprising Machine Learning (ML) methods offer various ways to design systems which improve human–machine interaction.

Prominent examples of such research lie in the fields of driver assistance and autonomous driving, which require reliable control interfaces to ensure passenger comfort and pedestrian safety. Ideally, a vehicular human–machine interface should empower drivers to interact intuitively with their in-car devices while staying focussed on the road ahead. To ensure that the drivers focus on the environment, a driver assistance system must require a minimum cognitive load to provide an advantage in safety and comfort. Freehand gestures, as means of easy non-verbal communication, apply well to this and similar situations.

For our research on hand gesture recognition systems, we aimed to develop a system to control an infotainment application, which runs on a mobile tablet computer mounted on the vehicles’ central console. To obtain a reasonable classification model, we started by examining support vector machines and multilayer perceptrons, which confirmed the advantage of deep learning technologies in terms of training and execution time. Our later deep learning approaches aimed to suit well for usage in smart mobile devices by lightness in model design.

Smart mobile devices, like current mobile phones and tablet computers, provide means of using different inputs, like touch or attached sensors but usually miss strong computational power, thus favouring lightweight implementations. Attaching ToF sensors allows us to receive hand gestures as depth data input in real time. Users usually quickly get accustomed to these smart devices, as they use them frequently in everyday life for communication, information and entertainment. A holistic hand gesture control system must meet the individual drivers’ expectations to provide a well-working interface that feels as natural and intuitive to the user as possible.

**Figure 1:**
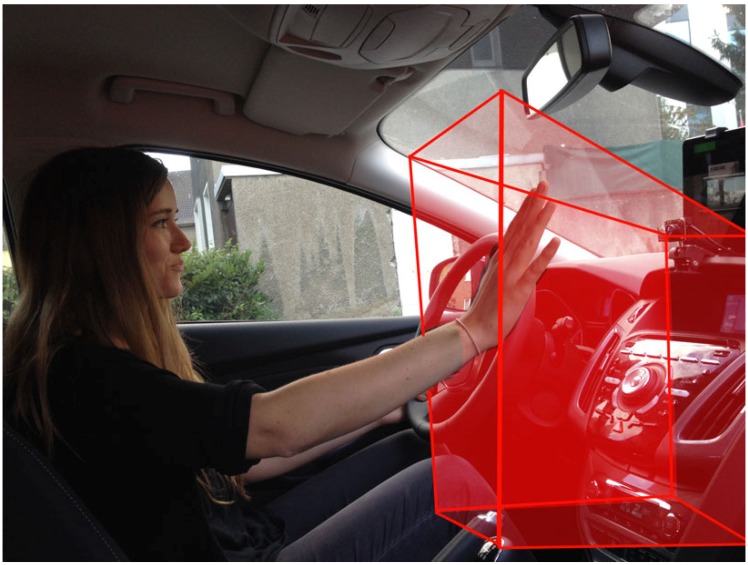
A driver performs a hand gesture in the detection range of time-of-flight (ToF) sensors (red area). This in-car setup uses a mobile tablet computer and two ToF sensors to recognise hand gestures in order to control an infotainment device.

Our in-car hand gesture recognition system makes use of one or more low cost ToF sensors attached to a mobile device, as shown in Figure 1. We recorded our data and performed our experiments using *Camboard nano* sensors, which provide depth images of resolution 165 × 120 px at a frame rate of 90 fps. These ToF sensors yield illumination independent depth data at a high frame rate. The extracted three-dimensional point clouds undergo a preprocessing procedure and serve as input for our investigated machine learning methods. Depending on the number of hand gestures to recognise and the input data dimensionality, a high-quality classification system demands robust preprocessing methods, adequate model parameters and fine-grained hyperparameter tuning to achieve high recognition rates in a low runtime.

To extract the full meaning of a given hand pose, we need to consider its temporal context; we make a conceptual distinction between dynamic hand gestures and static hand poses. A dynamic hand gesture unfolds in time, while a static hand pose does not. Therefore, a sample of a dynamic hand gesture consists of consecutive frames containing several hand poses and their transitions in a sequence. In our early experiments, we only recognised static hand postures with support vector machines and multilayer perceptrons, while in our later research we proceeded by identifying dynamic gestures with first an algorithmic machinery on top of a static hand posture recognition system and later a recurrent neural network architecture especially designed for dynamic hand gesture recognition.

We start this review by delineating the related work we based our research on and examining current state-of-the-art technologies in Section 2. In Section 3, we first introduce our data set for Recognition of Hand Postures (REHAP) and the underlying preprocessing method, then carry on by explaining our investigations in detail. A short comparison of all our models consolidates our results. We then close this section by illuminating the usability studies we conducted. In Section 5, we conclude and discuss this review, proposing future work in Section 5.1.

## 2. State of the Art

**Figure 2:**
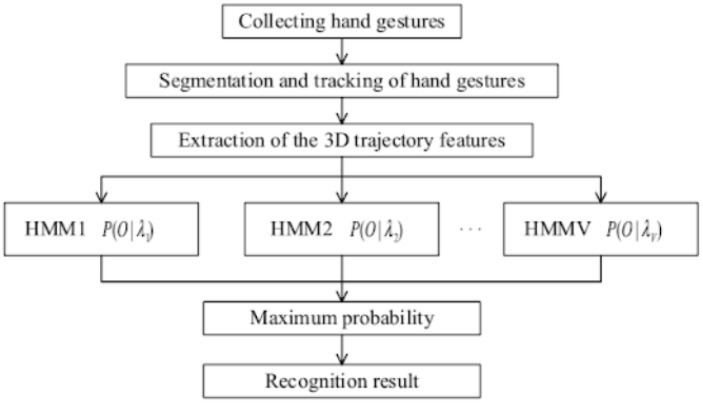
Dynamic hand gesture recognition flowchart from [5].

Related work in this field utilises a variety of different models and hand gesture datasets. In general, depth information helps to distinguish ambiguous hand postures, as described by [1], yet a lot of related work does not use deep learning methods on depth data, but provides valuable insights into various ideas of how to approach hand gesture recognition in other ways. For example, Ref. [2] provided an interesting study that uses electromyographic signals from wearable devices and deep transfer learning techniques to reliably determine a hand gesture with recognition rates up to 98.31%. For another example, Ref. [3] successfully implemented a recognition system which achieves up to 87.7% accuracy on the American Sign Language (ASL) and claimed it as “*the first data-driven system that is capable of automatic hand gesture recognition*” without any deep learning methods but hidden Markov models (HMM). Similiarly, Ref. [4] proposed aggregated HMM in a gesture spotting network (GSN) for navigating through medical data during neurobiopsy procedures. Their contribution achieves a recognition rate of 92.26% on a set of dynamic gestures like hand waving, finger spreading and palm movements. Ref. [5] contributed an improved dynamic hand gesture recognition method based on HMM and three-dimensional ToF data, as sketched in Figure 2. The authors use an adaptive segmentation algorithm for hand gesture segmentation, combining a frame difference method with a depth threshold. A hand gesture recognition algorithm based on HMM then takes full advantage of the depth data. In order to improve the recognition rate of the dynamic hand gesture, the authors feed the misclassified samples back into training. The authors report high recognition rates around 95% on dynamic hand gestures with robustness to the different backgrounds and illumination.

Refs. [6,7,8] used the Kinect camera for hand gesture recognition purposes, operating simultaneously on RGB and depth data. On a minimal example of 75 data points, Ref. [9] managed to obtain a 100% recognition rate by applying first a depth threshold, then contour image algorithms and naïve Bayes classification. Ref. [10] achieves 94% accuracy on the ASL dataset with convolutional neural networks that operate on RGB image sequences only.

Inspired by [11], we isolated the relevant hand part from the rest of the body by a simple depth threshold in some of our early experiments and used Principal Component Analsysis (PCA) later on. During our early stage of experiments, state-of-the-art algorithms achieved good performances but only on very limited datasets, or if designed for a specific application, as pointed out by [12]. Ref. [13] used a single ToF-sensor and employed the Viewpoint Feature Histogram (VFH) descriptor to detect hand postures, which confirms the importance of appropriate point cloud descriptors. Improved results, as achieved when fusing stereo camera information from depth sensors, for example by [14], lead us to confirm the advantage of using a second sensor and performing sensor fusion.

Dynamic hand gesture recognition poses the problem of spatiotemporal segmentation as pointed out by [15], who proposed a modular framework to solve this problem. Some but not all colour image based approaches rely on the detection of certain hand pixels [16] and employ algorithms or finite-state machines to detect dynamic gestures [17]. Our contributions differ in that we approach this problem solely data-driven with three-dimensional depth images.

Ref. [18] presented a gesture recognition system that also operates on ToF depth data only, which proves to save on computational cost. To avoid wearing special devices, the authors proposed a new algorithm that computes the wrist cutting edges, captures the palm areas and performs finger detection to judge the number of fingers, which significantly reduces the usage of computational resources. Their method achieves a recognition rate of 90% on their dataset.

**Figure 3:**
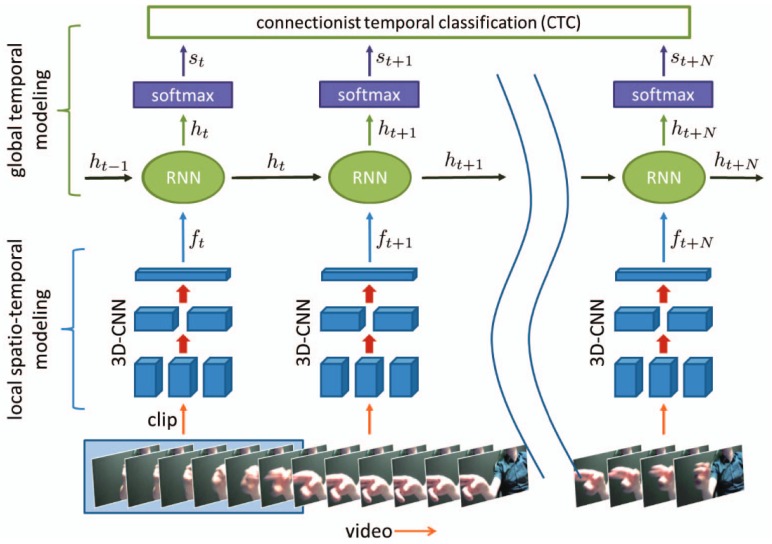
The recurrent three-dimensional convolutional architecture from [23]. As input, the network uses a dynamic gesture in the form of successive frames. It extracts local spatio-temporal features via a 3D Convolutional Neural Network (CNN) and feeds those into a recurrent layer, which aggregates activation across the sequence. Using these activations, a softmax layer then outputs probabilities for the dynamic gesture class.

In the last several years, a lot of hand gesture recognition benchmark datasets emerged. Ref. [19] published a dataset containing a total of 3,000,000 frames of 24,000 egocentric gesture samples with both colour and depth information, sampled from 50 distinct subjects. Another dataset, the Cambridge gesture recognition dataset, contains nine hand gesture classes sampled from two persons in 100 video sequences in five different illumination setups [20]. Ref. [21] reports an accuracy of 95% by employing a long-term recurrent convolution network to classify dynamic hand gestures from the Cambridge gesture recognition dataset. Their system extracts most relevant frames by a semantic segmentation-based deep learning framework, which represents the relevant video parts in the form of tiled patterns. Ref. [22] published the ChaLearn IsoGD dataset, which contains a total of 1000 object classes and approximately 1,200,000 training images, including 249 gesture labels and 47,933 manually labelled dynamic hand gesture sequences with RGB-D information. Ref. [23] investigated a three-dimensional convolutional neural network with a recurrent layer, shown in Figure 3. To validate their method, the authors introduced a new dynamic hand gesture dataset captured with depth and colour data, referred to as the Nvidia benchmark in later research. On this dataset, their gesture recognition system achieved an accuracy of 83.8%.

**Figure 4:**
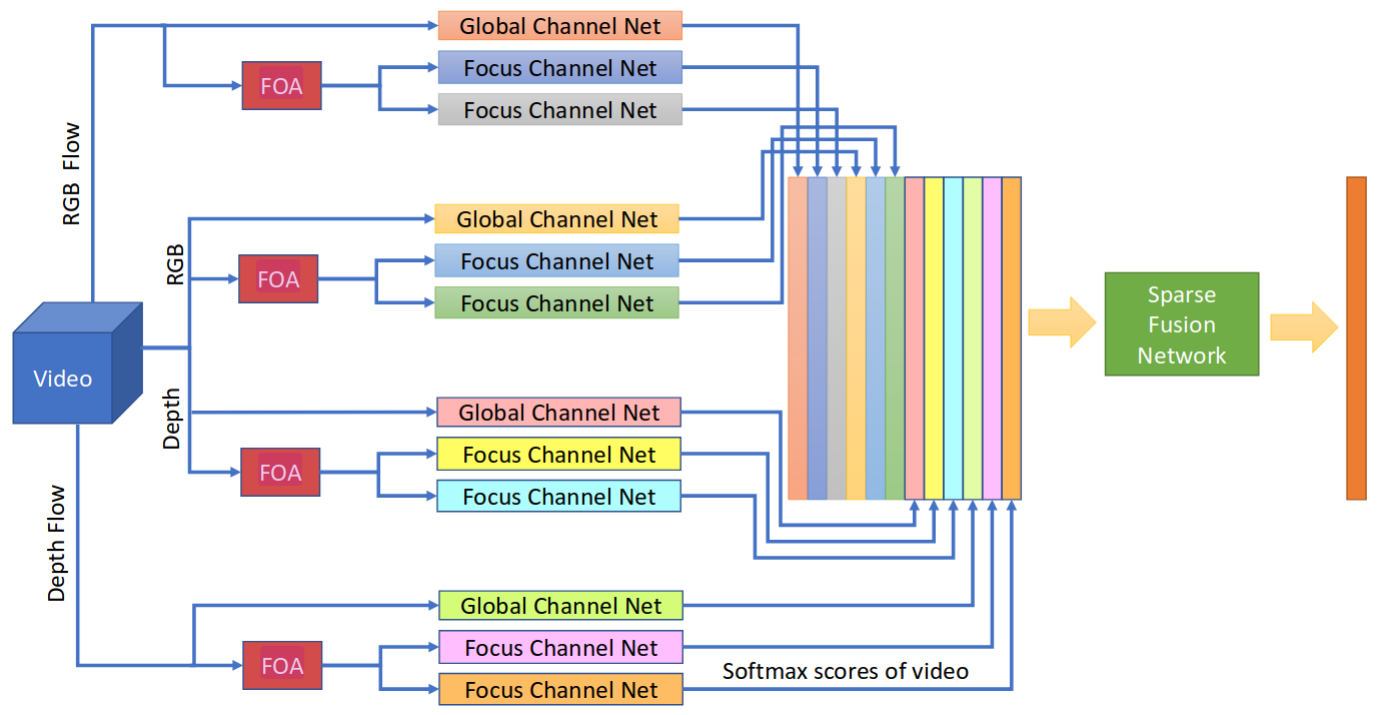
The FOANet architecture [24], which consists of a separate channel for every focus region (global, left hand, right hand) and input modality (RGB, depth, RGB flow and depth flow).

Figure 4 shows that distributed spatial focuses on the hands improved gesture recognition, especially when using a sparse network fusion technique. Using their FOANet architecture, depicted in Figure 4, the authors improved the performance on the ChaLearn IsoGD dataset from a previous best of 67.71% to 82.07%, and on the Nvidia dataset from 83.8% to 91.28%, even though the FOANet does not make use of any temporal fusion but optical flow fields of RGB and depth images. Their architecture consists of a separate channel for every focus region and input modality. An integrated focus of attention module (FOA) detects hands, a softmax score layer stacks 12 channels together and a sparse fusion layer combines the softmax scores according to the gesture type probabilities. With that architecture, the authors surpass both the previous best result and human accuracy, as shown in Table 1. The accuracy of FOANet drops when replacing sparse network fusion by average fusion. From the results listed in Table 2, we can see that the focused RGB flow field channel performs the best. In addition, we observe a general advantage of focus channels compared to global channels.

Concerning user interface design, as important in our later user studies, Ref. [25] investigated and compared different menu designs. Ref. [26] gives an overview on important aspects of good user interface design for automotive environments. The question arises what makes up an intuitive user interface in the context of human–machine interaction. Ref. [27] reminded readers that users need time to grow accustomed to any new devices; the authors mentioned that most users did not consider the computer mouse an intuitive device on first encounter.

In a literature review on hand gesture recognition with depth images, Ref. [28] studied 37 papers with a total of 24 methods. Ref. [29] presented a comprehensive overview of relevant basic ideas for hand gesture recognition, concerning computer vision in general and various machine learning techniques in particular. Ref. [30] contributed an overview on several methods leading to good results, which depend on the concrete application setup. Ref. [31] reviewed literature on various gesture recognition methods including neural networks, hidden Markov models, fuzzy c-means clustering and orientation histograms for features representation. More literature reviews, as Refs. [32,33], convey further information about hand gesture recognition with depth data.

## 3. Implementations

**Figure 5:**
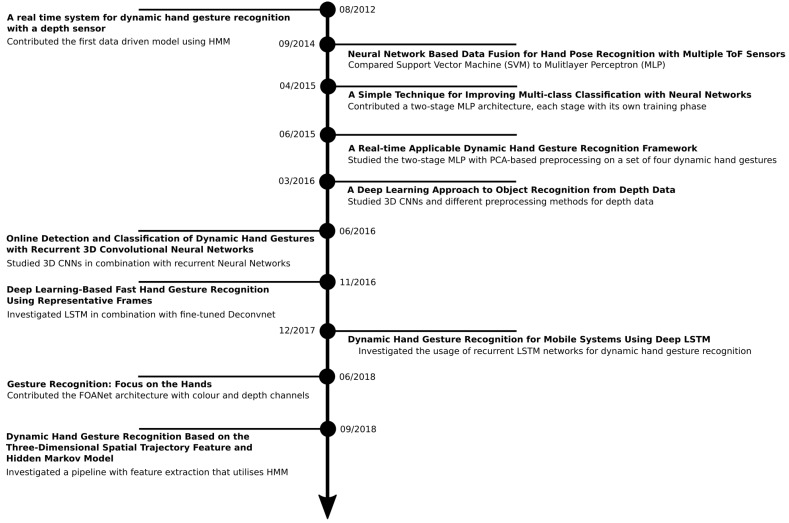
The timeline consolidates some but not all important contributions in the field of hand gesture recognition with depth data. On the right-hand side, we name the important contributions of our own line of research; on the left-hand side, we show the most relevant studies from other research teams.

**Figure 6:**
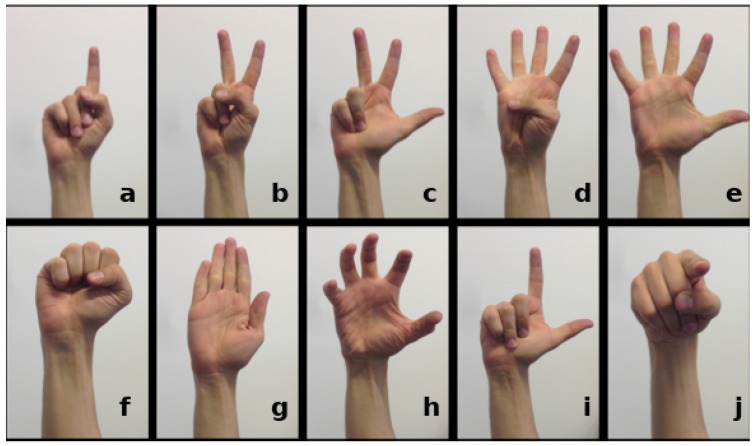
The set of ten hand posture classed (**a**–**j**) that we used throughout our research and provided samples from in our REHAP if appropriate benchmark dataset [34]. The hand poses in the top row raise different numbers of fingers, indicating a certain numerical value. The classes in the bottom row feature other meaningful postures like a fist, a flat hand, a grip, an L-shape and finger pointing, which form some elements for dynamic hand gestures.

In this section, we consolidate the line of our own contributions, starting with an introduction of our REHAP dataset and the underlying preprocessing method. We then examine our line of deep learning research on hand sign recognition from the beginning to the current state and summarise the results. At the end of this section, we explain the user studies we have performed in order to assess the usability of our system in practice. The timeline in Figure 5 shows some but not all important contributions in the field of hand gesture recognition to which we relate our contributions. The same set of ten hand pose classes, as depicted in Figure 6, had underlain our whole course of research.

### 3.1. REHAP Dataset

**Figure 7:**
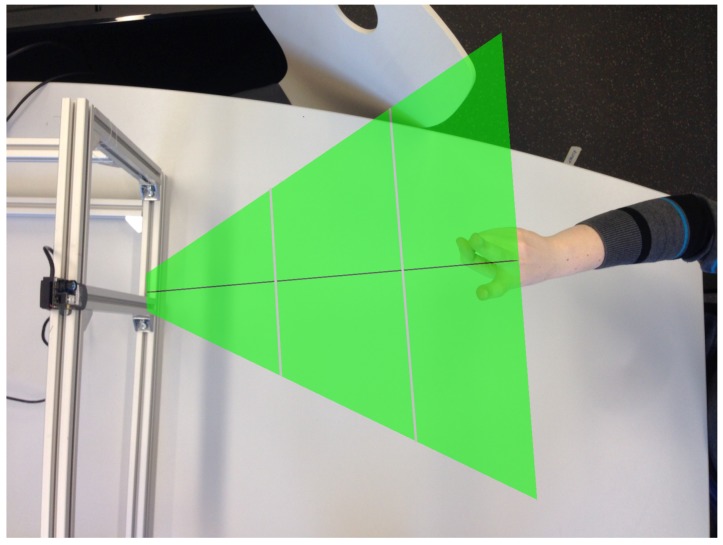
A setup for recording hand gestures with a *Camboard Nano* sensor. We divide the detection area into three zones: near (15–30 cm), intermediate (30–45 cm) and far (45–60 cm).

**Figure 8:**
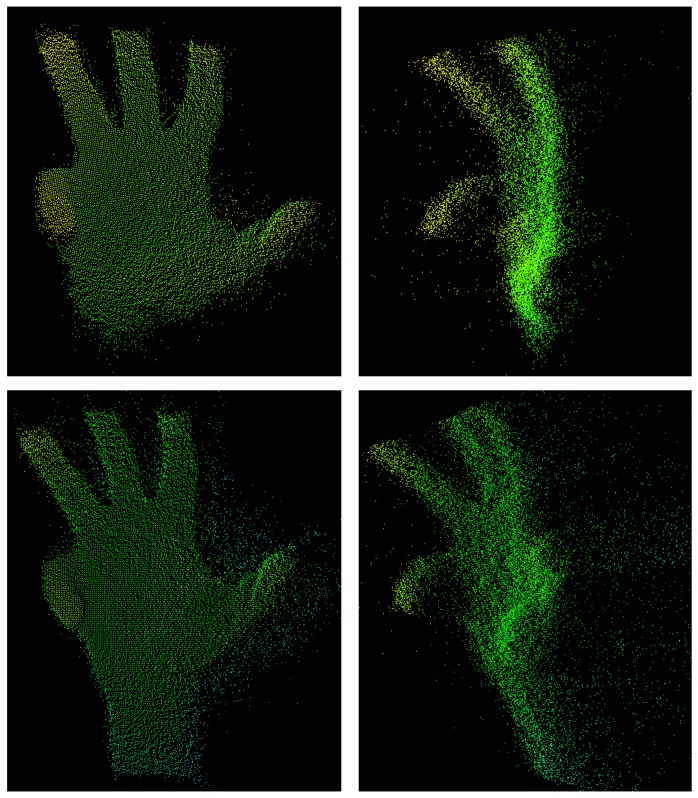
A raw three-dimensional point cloud of a grip-like gesture. (**left** and **right** column): the same gesture from different points of view; (**top** row and **bottom** row): the same gesture a few frames later.

As we proceeded in our course of research, we gathered the hand gesture depth data to provide a common basis for data-driven training. Ref. [34] published the REHAP dataset (Recognition of Hand Postures, REHAP, [35]). The whole dataset contains over a million unique samples of our ten hand postures shown in Figure 6 and consists of two disjunct parts: 600,000 sample images from 20 different persons (REHAP-1) and 450,000 samples from 15 different persons (REHAP-2). Depth images in REHAP-1 feature a resolution of 160 × 120, resulting in a point cloud size of 19,200 before cropping. The images in REHAP-2 have a doubled resolution of 320 × 240. The data aggregated in our REHAP-1 dataset consists solely of ToF sensor depth images, while the dataset REHAP-2 also contains corresponding RGB images. In contrast to some other hand gesture datasets, REHAP also provides images sampled from different points of view. We sampled our data with a setup as shown in Figure 7 in the form of point clouds, as depicted in Figure 8.

#### PCA-Based Preprocessing

**Figure 9:**
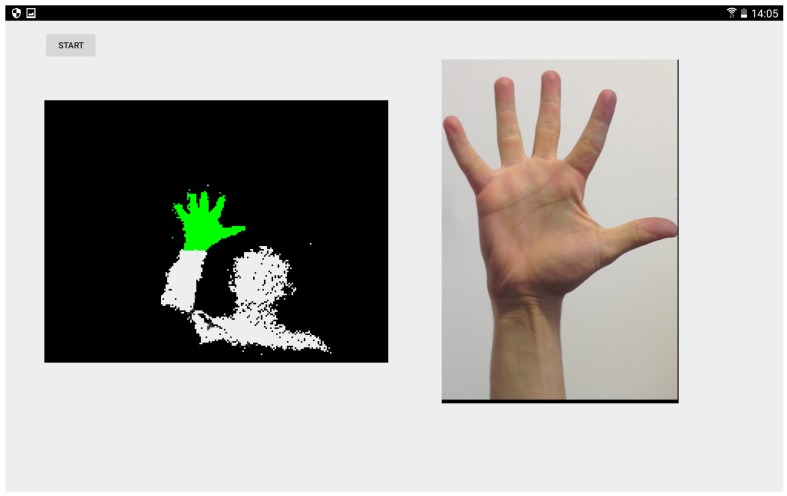
A demonstration of our preprocessing method. After applying a depth threshold and Principal Component Analysis (PCA), the green voxels (**left**) remain for further processing and serve to identify the correct hand posture class (**right**).

**Figure 10:**
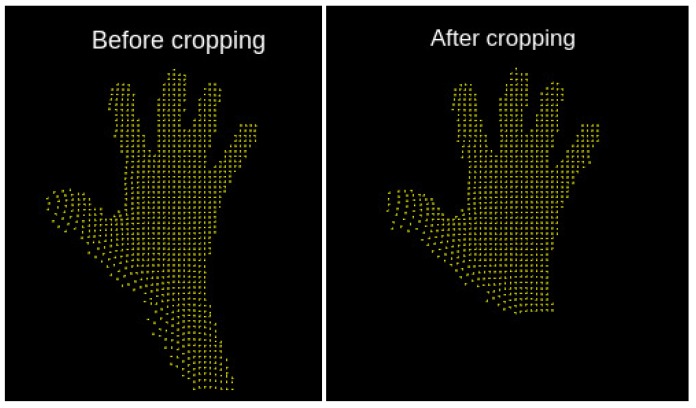
Principal component analysis of a raw hand posture point cloud (**left**) effectively performs cropping to the essential hand parts (**right**), which contains the most relevant information for hand posture classification.

Data points belonging to the forearm carry no relevant information concerning the hand posture class, so we used Principal Components Analysis (PCA) to crop our data to the essential part, as shown in Figure 9 and Figure 10. Ref. [36] contributed a preprocessing method using principal component analysis (PCA) to effectively crop the data, such that it only contains the palm and the fingers. In contrast to other possible cropping procedures, as recurrent neural networks or similar models, the strength of PCA lies in the fact that this unsupervised machine learning methods needs no training and operates fast, using only lower order statistics.

PCA reduces the dimensionality of high-dimensional data, like our point clouds, by linearly projecting them onto a lower-dimensional subspace. It estimates the direction of largest variance; in our case, the essential hand pose part of the depth image, by solving the eigenvector equation for the depth images’ covariance matrix. For an input vector *x* with *n* three-dimensional coordinates, we compute a mean value $\bar{x}$ as:

$$\bar{x}=\frac{1}{n}\cdot \sum_{j=1}^{n}\left(x_{j}\right),$$

and continue by estimating a covariance matrix as scatter matrix *S*:

$$S=\sum_{j=1}^{n}\left(x_{j}-\bar{x}\right){\left(x_{j}-\bar{x}\right)}^{⊤}.$$

Solving the eigenvector equation for *S* and keeping only the eigenvectors with the largest eigenvalues leaves us with the vector containing most information, the principal components.

### 3.2. Investigated Methods

In this section, we describe our investigations in detail. Ref. [37] started by conducting hand gesture recognition experiments using two ToF sensors, comparing different 3D point cloud descriptors and testing a multilayer perceptron versus a support vector machine (SVM). Different point cloud descriptors exist; in our research, we studied the Ensemble of Shape Functions (ESF, [38]), the Point Feature Histograms (PFH, [39]) and the Viewpoint Feature Histogram (VFH, [40]), implemented in the Point Cloud Library (PCL). Then, Ref. [41] proposed a simple technique to boost classification performance of a multilayer perceptron by adding a second multilayer perceptron, which feeds on the output of a first multilayer perceptron (MLP) as well as the original input. Ref. [42] designed a three-dimensional convolutional neural network which outperformed the previous approaches in static hand pose recognition. Ref. [43] employed a recurrent long short-term memory network in order to classify upon a coherent sequence of frames of a dynamic hand gesture. Table 3 summarises all the final results.

#### 3.2.1. Early Investigations

In 2014, Ref. [44] examined the performance of support vector machines (SVM) for hand gesture recognition on depth data in detail. For our SVM research, we studied extensions of large margin classifiers as in [45,46,47,48] and multinomial kernel regression as in [49], focussing on multi-class decomposition as proposed by [50]. A one-versus-all (OVA) [51,52] approach trains a binary classifier to distinguish one class from all the other classes, whereas the one-vs.-one (OVO) approach [53,54,55,56,57,58,59] trains a binary classifier for each pair of classes, and more complex graph-based approaches [60,61] construct decision trees. For our course of research, we used the OVO approach.

**Figure 11:**
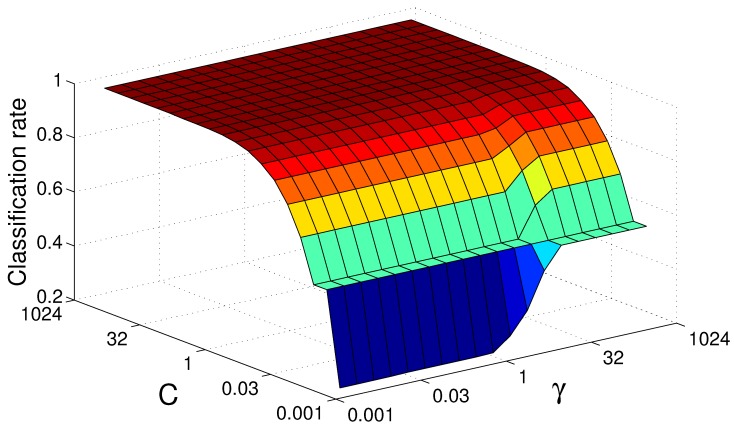
Support vector machine grid search landscape for Radial Basis Function (RBF) kernel parameters subject to the Viewpoint Feature Histogram (VFH) descriptor. For an increasing penalty factor *C*, the kernel parameter γ decreases in significance.

With a training time of approximately two days, the best SVM trial resulted in a classification accuracy of 99.8% with a Gauss kernel. We obtained an optimal SVM parametrisation by performing a grid search with crossvalidation, which took approximately 16 days. Figure 11 illustrates the parameter landscape for our Radial Basis Function (RBF) kernel parameter grid search using the VFH descriptor. The dataset used for training and testing contained a total of 320,000 samples on different angles, randomly split into two parts of equal size.

Later, Ref. [37] investigated two neural network based fusion strategies with a multilayer perceptron: the early fusion and the late fusion strategy. An early fusion approach classifies a hand posture given a vector of concatenated descriptors, while a late fusion strategy classifies each data point individually and later combines predictions. Both strategies proved to perform equally well.

To obtain a reasonably small descriptor size *K*, a preprocessing algorithm samples 20,000 points from the input point cloud at random and then continues by repeatedly sampling three random points, from which it calculates a descriptor histogram. For a descriptor histogram size *K* and *N* sensors, the multilayer perceptron in this experiment has an input layer of size N·K neurons, a hidden layer with 150 neurons and 10 output neurons, one for each class. To improve predictions, we probed three different confidence measures for post processing of output neurons activations $o_{i}$:

$$\begin{array}{cc} \mathrm{confOfMax}(\left\{o_{i}\right\}) & =\max\;o_{i},\\ \mathrm{diffMeasure}(\left\{o_{i}\right\}) & =\max_{i}\;o_{i}-\max_{i}^{2}\;o_{i},\\ \mathrm{varianceMeasure}(\left\{o_{i}\right\}) & =\frac{1}{N}\sum_{i}{\left(o_{i}-E\left(\left\{o_{i}\right\}\right)\right)}^{2}. \end{array}$$

Finally, the system decides the class as:

$$\begin{array}{c} \mathrm{class}=\left\{\begin{array}{cc} {\mathrm{argmax}}_{i}o_{i}, & \;\mathrm{if}\;\mathrm{conf}\left(\left\{o_{i}\right\}\right)>\theta_{\mathrm{conf}},\\ \mathrm{no}\;\mathrm{decision},\; & \mathrm{else}. \end{array}\right. \end{array}$$

**Figure 12:**
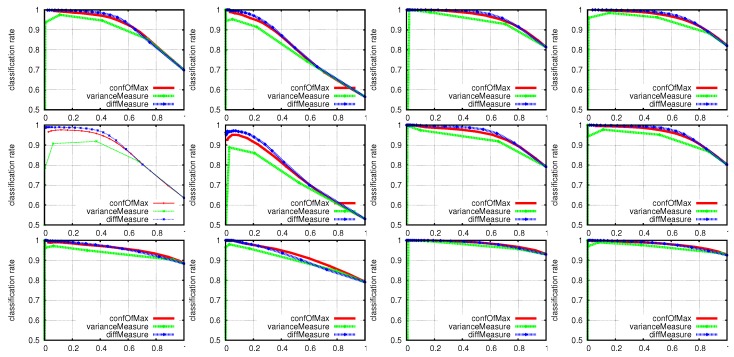
Classification rates for $\theta_{\mathrm{conf}}$ in the range of [0,1] for different descriptors and confidence measures using an MLP. The first row shows the VFH descriptor accuracies for ToF sensors in an angle of 30°, respectively in an angle of 90° for the second row. The third row concerns the ESF descriptor for an angle of 30° between the two sensors. From left to right, the first two columns show the performance when using only the first or second ToF sensor; the third column represents the late fusion approach and the fourth column the early fusion approach.

As Figure 12 points out, our confOfMax and diffMeasure confidence measures show little difference in performance compared to the varianceMeasure. Although the diffMeasure manifests a slightly superior curve, we use the confOfMax measure in further experiments with confidence thresholds in the range of [0.6,0.9].

Ref. [62] evaluated the impact of varying confidence measures and thresholds. The dataset in this experiment features a total of 400,000 samples. Our PFH descriptor exploits the fact that the tilt, pan and yaw angles describe rotation-invariant means of the alignment of two three-dimensional data points. We construct a histogram as proposed by [63] to further exploit this invariance by subsampling a certain number of points, calculating angular features and binning them into our histogram.

Testing various hidden layer sizes lead us to use an architecture in three layers of N·K=1250 input, 16 hidden and 10 output neurons. With an increasing confidence threshold value $\theta_{\mathrm{conf}}$, both the systems’ recognition accuracy and rejection rate increases, as illustrated in Table 4. For a high confidence threshold of $\theta_{\mathrm{conf}}=0.95$, we can state that the recognition rate raises close to 100%, but the system rejects about a third of all samples.

Ref. [41] proposed a general technique to boost classification accuracies of multilayer perceptrons. The idea advises to add a second MLP operating on the first MLPs output activation plus the original input. Evaluations on a hand gesture dataset, containing a total of 450,000 samples from 15 individuals, resulted in a classification accuracy improvement of about 3%. In our experiments, we have used 25 neurons in the hidden layer of our primary MLP and 20 neurons in the hidden layer of our secondary MLP. Table 5 shows the average increase or decrease in recognition rate for all ten gesture classes when adding the second MLP. Table 6 lists individual predictions accuracies of each MLP.

To investigate generalisation performance, as shown in Table 7, we asked 16 different persons to perform our ten hand poses and then trained a two-stage MLP to predict on one unknown person after learning based on the 15 other persons. With an overall generalisation performance of 77.0% the two-stage MLP with a hidden layer size of 50 neurons slightly surpasses all other models.

**Figure 13:**
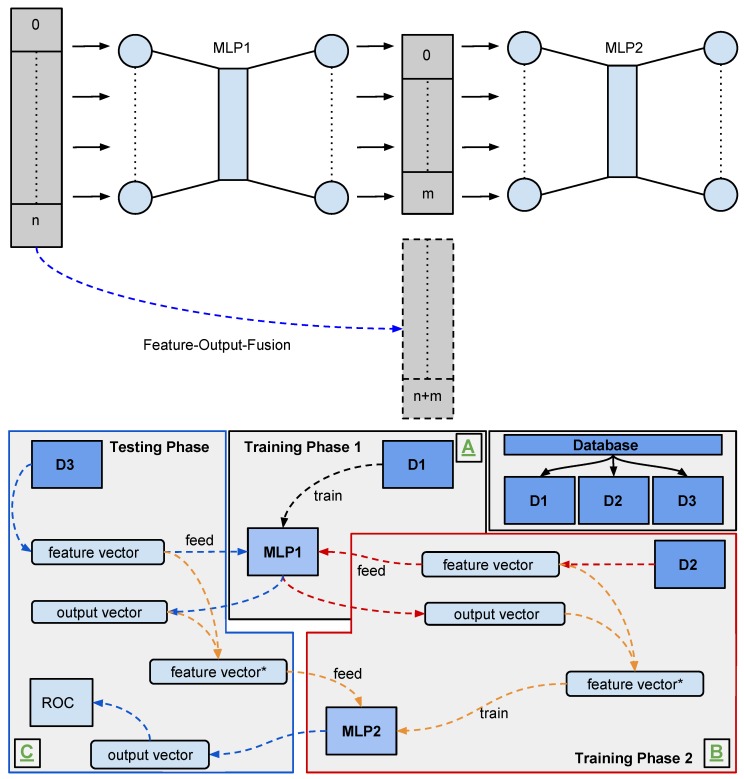
A sketch of our two-stage MLP model (**top**) and an affiliated training procedure (**bottom**). For an input histogram of size n=625 and an output layer size of m=10 neurons, the secondary MLP receives the primary MLPs’ output activation plus another histogram as input. In our training procedure, we split our dataset *D* in three parts $D_{1}$, $D_{2}$ and $D_{3}$, such that we may train our two MLPs on independent parts of the dataset and test it on unseen data.

**Figure 14:**
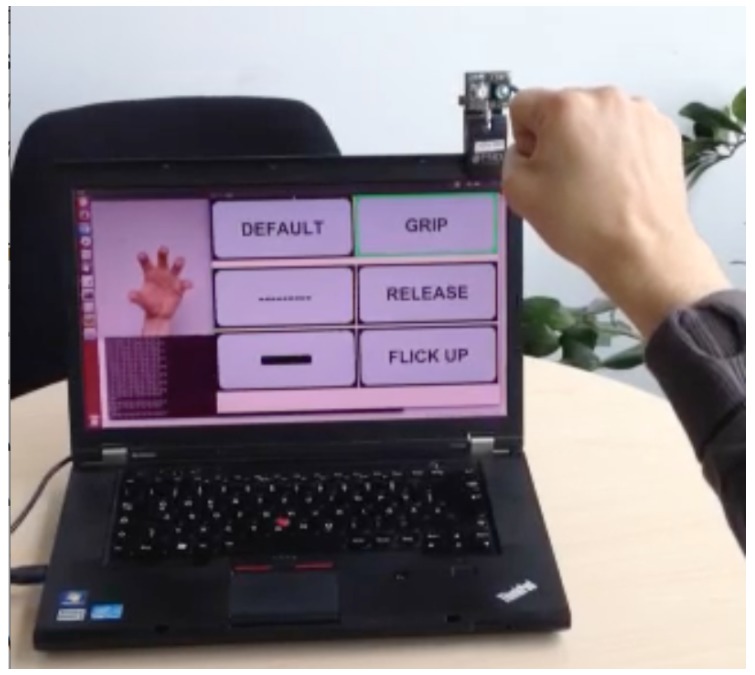
An exemplary hand gesture recognition setup. A user performs a dynamic gesture defined by a starting pose and an ending pose. For a dynamic grabbing gesture, pose class $s_{t}^{start}=h$ (grab) starts the sequence which $s_{t+m}^{end}=f$ (fist) ends. See Figure 6 for an overview of our ten hand pose classes. Our machine learning systems recognise the individual poses and an algorithm on top tries to identify the sequence.

Ref. [36] demonstrated an in-car dynamic hand gesture recognition system relying on a PCA-based preprocessing procedure. In these experiments, we considered dynamic hand gestures as listed in Table 8, each defined by a starting and an ending hand posture $s^{start}$ and $s^{end}$ as illustrated in Figure 14. A custom version PFH descriptor, which maintains real-time capability while gaining descriptive benefits, randomly chooses 10,000 point pairs and uses the quantised point features to build a global K=625-dimensional histogram. A two-stage MLP like the one depicted in Figure 13, implemented using the Fast Artificial Neural Network (FANN) library [64] with 50 neurons in each hidden layer, recognises a hand pose at each time step *t*. To recognise a dynamic gesture, we observe any occurrence of the starting state $s_{t}^{start}$ followed by any occurrence of the ending state $s_{t+m}^{end}$ with m≥1. This simple algorithmic approach allows for misclassification amidst the sequence without disrupting the recognition and results in an overall classification rate of 82.25% averaged over all persons and dynamic gestures, with a 100% recognition rate for zooming in, 90% for zooming out, 80% for release and 59% for grabbing.

#### 3.2.2. Convolutional Neural Network

**Figure 15:**
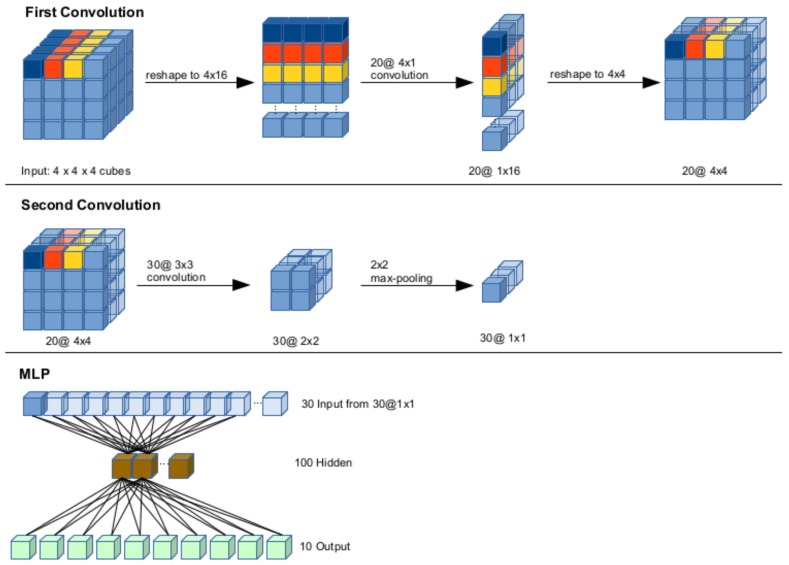
The structure of our CNN model. The first convolution step followed by a reshape (**top** row) yields input for the second convolution step, followed by a max-pooling layer (**middle** row), whose activation provides input for an MLP with 100 hidden neurons and 10 output neurons, one per hand pose class (**bottom** row).

Ref. [42] contributed a method to transform three-dimensional point cloud input into a fixed-size format suitable for convolutional neural networks (CNN) by studying three different approaches, each focusing on the subdivision and normalisation of a three-dimensional input point cloud. The first approach sums up the points within a cube (PPC), the second approach uses a two-dimensional projection of input cloud (2DP) and the third approach relies on least-squares plane fitting, calculating the normal of a plane per cube (NPC). Table 9 lists the performances we measured on our REHAP dataset as well as the data contributed by [65] for reference. Studies of these different approaches resulted in a preprocessing method we used for further experiments: first, we apply PCA to crop the input to the essential hand part. As our model expects the input space shape as $n^{3}$ hypercubes of fixed size, we normalise the point cloud to range (0,1) on each axis and then stretch it to fit into the raster. We reshape the input vector component-wise such that each component represents one slice of the original depth data. The CNN architecture, as depicted in Figure 15, requires an input matrix of 4×4×4 voxels. To obtain optimal parameters for the layers in our convolutional model, we performed a grid search on a set of parameters, as shown in detail in Table 10:

$$\begin{array}{cc} k_{i}^{1} & \in \left\{5,10,15,20\right\},\\ k_{j}^{2} & \in \left\{5,10,15,20,25,30,35,40,45,50\right\},\\ k_{s}^{1} & \in \left[0,7\right],\\ k_{s}^{2} & \in \left[0,7\right],\\ k_{mp}^{2} & \in \left[1,8\right]. \end{array}$$

**Figure 16:**
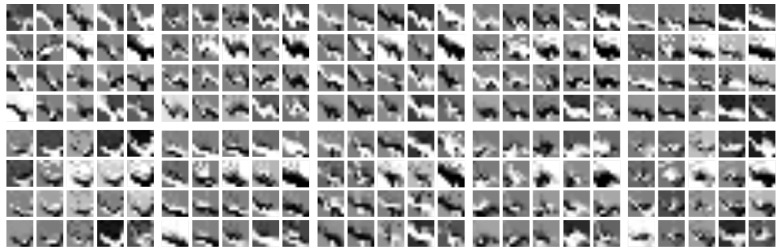
Resulting filter activations for the 20 kernels in the first layer of our CNN model from Figure 15. This figure shows the filtered activation for each hand gesture, grouped as in Figure 6.

$k_{i}^{1}$ and $k_{j}^{2}$ denoting the number of kernels within respective layers, $k_{s}^{1}$ a specific combination for the first layer, $k_{s}^{2}$ the size of the second kernel and $k_{2}^{mp}$ the kernel size in the max-pooling layer. At this stage of research, our REHAP dataset consisted of 600,000 samples, yet we chose to reduce the amount of data samples during training to about 2000 samples per gesture, each randomly taken from the whole sample set. This still yields a training set of 380,000 samples, from which we took 70% for training and 30% for testing. Ref. [66] reports best classification error scores achieved in this grid-search around 5.6%, averaged across all samples. Achieving classification rates up to 98.5%, with an average classification error of about 16%, the CNN outperforms our previous approaches on the REHAP dataset in terms of generalisation capability. Figure 16 shows the kernel activations that emerged after training.

#### 3.2.3. Long Short-Term Memory

Ref. [43] presented a hand gesture recognition pipeline for mobile devices using a deep long short-term memory (LSTM) network. As our our REHAP dataset does not contain dynamic gesture sequences but static frame, we recorded new data. For training and testing, we used a small dataset of only four hand gesture classes (close hand, open hand, pinch-in and pinch-out). Again, we recorded with a *Camboard Nano* ToF sensor at a resolution of 320 × 160 pixels and preprocesses the data with our PCA method, such that the network input consists of a 625-dimensional histogram. For a single dynamic gesture recording, we collected 40 consecutive snapshots. We gathered a total of 150 dynamic gesture samples of four classes, each with 40 frames per gesture, summing up to a total of 24,000 data samples. From this data, we used $N_{train}=480$ samples for training and $N_{test}=120$ for testing.

Using a standard LSTM model with forget gates as described by [67], we perform a grid search to obtain the optimal model batch size B∈{2,5,10}, memory block M∈{1,2,3,4}, LSTM cells per memory block C∈{128,256,512}, number of training iterations $I\in \{10^{3},5\times 10^{3},10^{4}\}$ and learning rate $\eta \in \{10^{-1},10^{-3},10^{-4},10^{-5}\}$. As the stochastic gradient optimiser, we employ the Adaptive Moment Estimation (ADAM) [68,69]. Measuring the generalisation performance ξ as the percentage of correctly classified testing labels, we found several parameter combinations achieving high recognition rates as shown in Table 11. For $P_{i})$ denoting the prediction for sample *i*, we computed ξ as:

$$\begin{array}{cc} {\tilde{p}}_{i} & =argmax(P_{i}),\\ \xi & =100\cdot \frac{\#({\tilde{p}}_{i}=l_{i})}{N_{test}}. \end{array}$$

**Figure 17:**
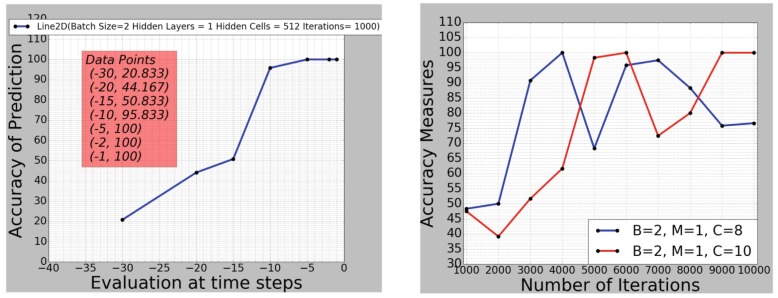
(**left**): accuracy of LSTM prediction on a single test data sample, with B=2, M=1, C=512 and I=1000, at different in-gesture time steps *t*; (**right**): accuracy of prediction taken at the end of a gesture, depending on training iterations for a small LSTM network size.

As our recurrent LSTM implementation aggregates activation across the temporal dimension of the sequence, the networks prediction accuracy gets more accurate as more frames it sees. Figure 17 shows an increase in performance plotted over the sequence’s temporal dimension. From this, we might expect an increase in confidence of classification for an increasing *t*.

The conducted experiments concern execution speed on a mobile device, generalization capability and predictive classification ability ahead of time. The recurrent architecture learns a label for a temporal sequence, which allows us to recognise a dynamic hand gesture already by the first few frames. As we do not need to wait for completion, our system operates with a natural advantage in execution time. Thus, high recognition rates above require less than 1ms computation time to detect a dynamic hand gesture.

## 4. Results

We state that the neural network outperforms an equivalent SVM implementation in terms of training and execution time, thus making it the better choice for a real-time driving scenario [37]. We show that the usage of a second ToF sensors improved results tremendously compared to using only a single sensor [37]. Ref. [42] demonstrates that convolutional neural networks show superior performance compared to previous approaches. Research on recurrent LSTM architectures promises high recognition rates and fast processing [43]. Table 12 summarises our results for the best known choice of hyperparameters, evaluation modalities and eventual post processing parameters.

The choice of a high confidence threshold leads to higher test performances compared to the training performance of our simple MLP. For the two-stage MLP, we split our dataset into three parts, from which we used the first two to train each stage separately. The high test results of the CNN architecture refer to the best test trial. As we recorded a new set of dynamic hand gestures for our LSTM network, we had only little data for these experiments.

### 4.1. Usability Evaluation

**Figure 18:**
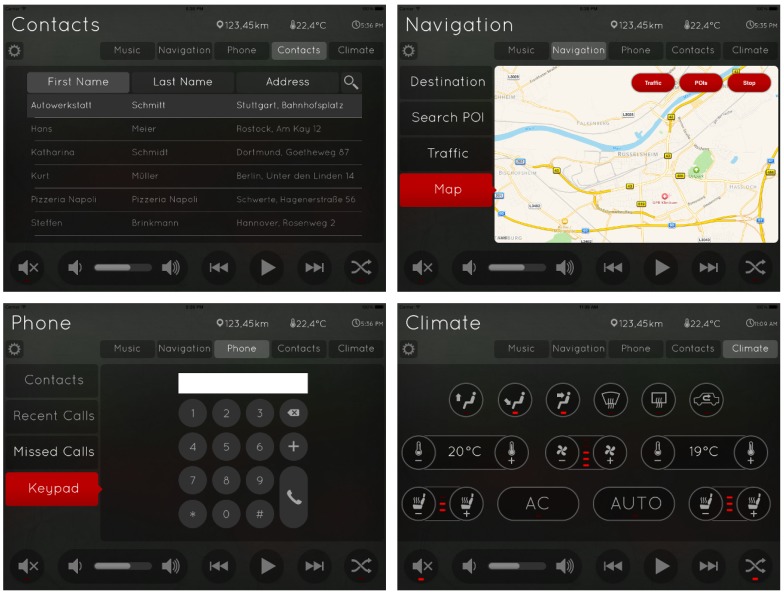
Sample screens of a dummy interface design used in our usability studies. The user may not address all functions via hand gestures but may navigate through the menus (social contacts, street maps, phone calls and in-car climate control) with gestures *a* to *e* from Figure 6 as well as start/stop (gesture *g*, *j*). In later versions, we also implemented controls for zooming.

To compare our freehand gesture recognition systems with traditional touch control, we performed intuitiveness studies with an INTUI questionnaire study [70,71] and a standardised Lane Change Test as explained in this section. Figure 18 shows the graphical user interface used in the evaluation experiments. We did not implement all screen controls for these tests but focus on using our ten freehand gestures for navigating the menu screens. Specifying the GUI design to provide a basis for examining the usability of our system, we did not implement the complete screen control but show that our ten hand postures suffice the requirements of our usability studies.

Ref. [72] published the results of a user study with an INTUI questionnaire [70,71], which aimed to measure our systems intuitiveness from the user perspective. Ref. [73] studied the Lane Change Test (LCT), as described in the ISO 26022 standard, to quantify the drivers distraction when interacting with our system via touch or freehand gestures.

#### 4.1.1. INTUI Studies

**Figure 19:**
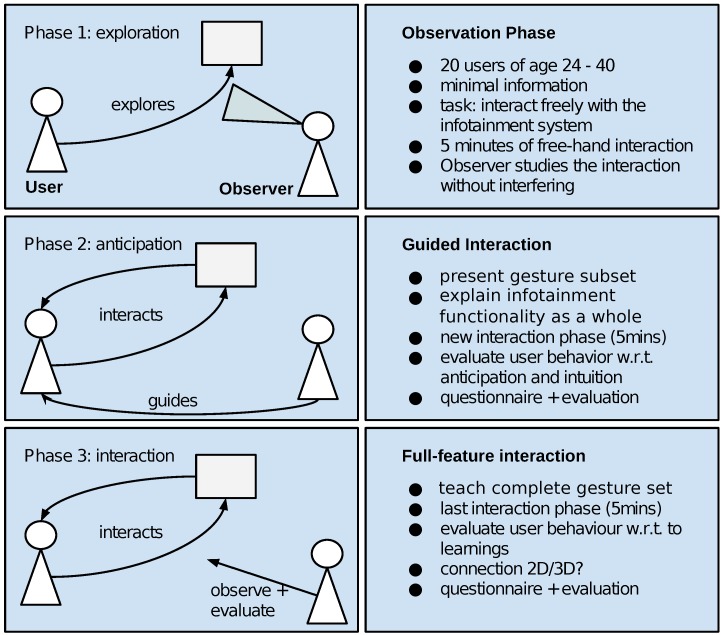
The procedure of our human–machine interaction experiment in three phases. Participants interacting with an in-car infotainment device start with a free exploration and later receive instructions on how to actually use the system.

In 2016, Ref. [72] published the results of a user study investigating our systems intuitiveness in an in-car human–machine interaction setting. In an experiment consisting of three consecutive phases, as summarised in Figure 19, a total of 20 participants interacted with the infotainment device via freehand gestures. Participants then answered an INTUI questionnaire [70,71], which tries to capture different aspects of intuitive interaction. During the experiment, we observed all participants trying to interact with dynamic gestures at the beginning. After receiving explanation of our hand gesture symbolism, all our participants managed to purposefully interact with the system. Relevant INTUI scores, as shown in Table 13, argue for a reasonably well overall acceptance of our system, considering initial frustration in phase one. As we observed all 20 participants trying to intuitively transfer gestures they knew from other gesture interaction systems, we state that a straightforward implementation of a naïve gesture recognition system does not lead to an intuitive human–machine interaction interface.

#### 4.1.2. Lane Change Test

**Figure 20:**
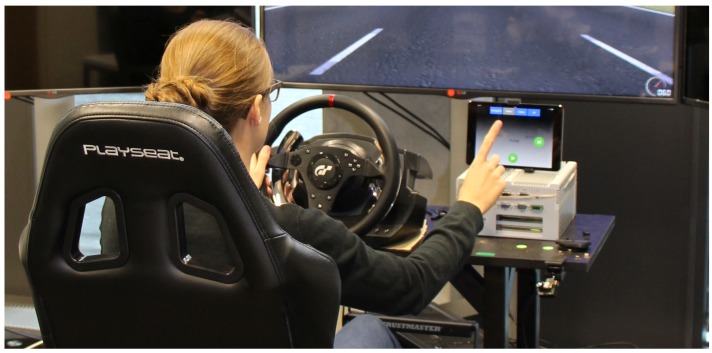
A participant performs the Lane Change Test while controlling the infotainment system via mid-air hand gestures.

**Figure 21:**
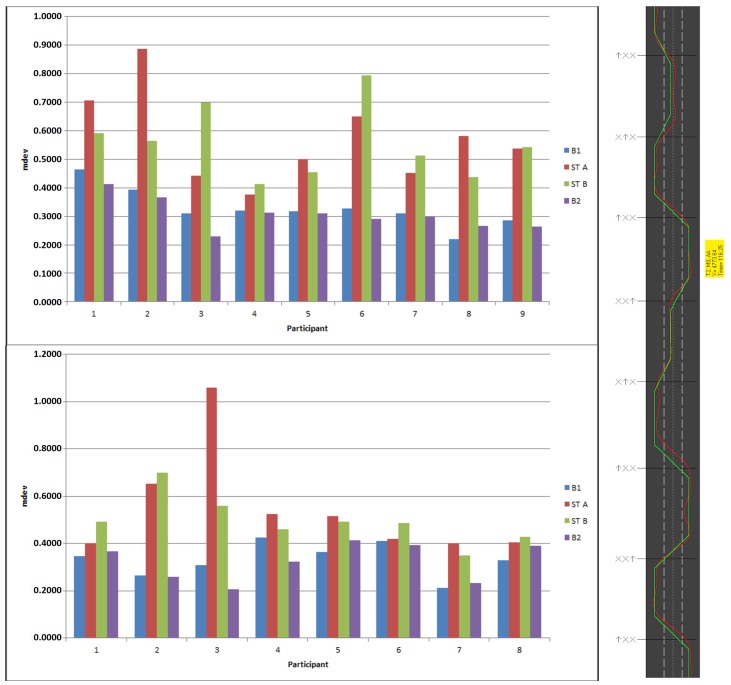
(**left**): Lane Change Tests results for the nine participants in group *T* (**top**) and the eight participants in Group *M* (**bottom**) in terms of mean deviation from the baseline, (**right**): an exemplary Lane Change Test track. The green line (baseline) shows the optimal course, the red line the trajectory actually driven by the participant. A trial lasts for approximately three minutes and contains a total of 18 lane change signs.

Ref. [73] performed the Lane Change Test (LCT), as described in the ISO 26022 standard and shown in Figure 20 and Figure 21. As LCT presumes a perfectly reliable recognition system, which we could not provide at that time, we conducted this study in a Wizard-of-Oz setup. This test aimed to measure the impact of freehand gesture interaction as a secondary task (ST) accompanying a primary driving task (PT) in terms of human driving performance. To complete the primary task, a participant drives a simulated vehicle on a predefined course with three lanes at a fixed velocity of $60\times 10^{3}\frac{m}{h}$. The secondary task requires the participant to redirect his or her attention to interact with an infotainment system either via touch control (ST A) or mid-air hand gestures (ST B). We measure the drivers distraction via the vehicles mean deviation (mdev) from an optimal course as shown in Figure 21 on the right-hand side. With a total of n=17 participants, all licensed drivers aged from 23 to 44 years, we performed our experiments in two groups: group *T*, with nine participants, started by controlling the infotainment system with touch gestures and later switched to mid-air hand gesture control, while group *M* with eight participants began with mid-air hand gestures and then used touch control. As proposed by the ISO standard, we choose four out of the ten available tracks at random and explain the hand gestures to the participant. The first of the four trials yields a baseline performance (B1) and the second trial estimates a learning effect via a second baseline (B2). Overall, four of our 17 participants from group *T* missed a traffic sign, resulting in a large mean deviation from the ideal route. Removing these four participants reduces the mdev scores for the secondary tasks to 0.49 for touch gesture interaction and 0.50 for mid-air gestures, respectively. After having performed the LCT, participants mentioned they perceived memorising the hand gestures as a high effort. Aside from the fact that both secondary tasks strongly influence the primary task performance, the results in Figure 21 show a slight advantage of touch gesture interaction in terms of driver distraction. This may come from the fact that the participants did not know any of the freehand gestures and need more learning time to use them as an intuitive mean of interaction.

## 5. Conclusions

In this review, we examined current state-of-the-art deep learning technologies for hand gesture recogniton and consolidated a line of research from the Computational Neuroscience laboratory at the Ruhr West University of Applied Sciences. Kopinski’s contributions [34,36,37,41,42,43,44,62,66,73,74,75,76,77,78,79] and PhD thesis [72] form the basis of our hand gesture recognition research. We investigated deep learning technologies for the purpose of hand gesture recognition in automotive context with three-dimensional data from time-of-flight infrared sensors in order to provide new means of controls for driver assistance systems. Comparing our approaches with related work, we state that our lightweight implementations suit mobile computing and feature reasonable accurarcy. With an INTUI questionnaire, we tried to assess the individual drivers feeling of familiarity when using our system the first time. A standardised Lane Change Test, as described by the ISO 26022 standard, illuminated the impact of our technologies on motorists driving behaviour. We have published our hand posture dataset (REHAP) as well as the source code, which compiles with a standard GNU Compiler Collection (GCC) and a make program under Ubuntu, at [35].

### 5.1. Future Work

Future work may examine the generalisation capability of our LSTM approach with larger datasets. In addition, we may combine our convolutional architecture with the recurrent layer and search for improvements in generalisation performance. To assess transfer learning capabilities, future experiments may try to transfer knowledge from our hand gesture symbolism into other hand sign languages.

We did not compare our freehand gesture traditional control mechanism like on-wheel buttons, an important comparison which we leave for later research. In addition, more user studies may yield a more detailed picture which parts of the system we might change to achieve a more intuitive human–machine interaction. Especially, a lane change test with participants trained our hand gesture symbolism might provide more realistic insights into advantages of freehand gesture control.

Given more computational resources, models with larger parameter spaces may perform slightly better, but such a comparison remains for future research. In general, future research may perform uniform comparisons across the whole zoo of hand gesture recognition systems and their respective datasets that emerged in the last years.

## Figures and Tables

**Table 1:** Overall results of FOANet and compared architectures on the Nvidia benchmark.

Method	Channels	Accuracy
FOANet	FOA + Sparse Fusion	91.28%
FOANet	FOA + Avg. Fusion	85.26%
Human	Colour	88.4%
Molchanov [23]	All (including infrared)	83.8%
Molchanov [23]	Depth + Flow	82.4%

**Table 2:** Individual channel performances of FOANet on the Nvidia benchmark.

	RGB	Depth	RGB Flow	Depth Flow
Global	43.98%	66.80%	62.66%	58.71%
Focus	58.09%	73.65%	77.18%	70.12%

**Table 3:** Support Vector Machine (SVM) classification accuracies for both kernel types, the ESF and VFH descriptors and two camera set ups in an angle of 30° respectively 90°.

Descriptor	ESF 30°	ESF 90°	VFH 30°	VFH 90°
Classif. rate scalar kernel	98.7%	98.8%	96.9%	94.2%
Classif. rate gauss kernel	99.8%	99.6%	98.8%	93.1%

**Table 4:** The impact of an increasing $\theta_{\mathrm{conf}}$ (top row) on the average classification error (middle row) and the number of rejected samples (bottom row), averaged over 100,000 samples.

*θ*_conf_	0	0.65	0.95
Avg. Error	6.3%	3.6%	1.3%
rejected samples	0	6776	34005

**Table 5:** The average change in classification accuracy for each of our ten hand gesture classes when using the two-stage model. Some but not all classes improve reasonably well, while class *d* seems to suffer in terms of recognition accuracy.

a	b	c	d	e	f	g	h	i	j
+0.01	+0.02	+0.01	−0.03	+0.07	+0.01	+0.05	+0.04	+0.01	+0.04

**Table 6:** Generalisation performance comparison of the primary Multilayer Perceptron (MLP) (upper row) and the secondary MLP (lower row) using the training procedure depicted in Figure 13.

	a	b	c	d	e	f	g	h	i	j
MLP1	90%	90%	89%	87%	87%	95%	92%	92%	89%	95%
MLP2	93%	93%	93%	90%	91%	97%	94%	94%	91%	96%

**Table 7:** Generalisation results for all 16 persons and both MLPs, each with 30 and 50 neurons, respectively. Here, column 1 represents the performance of our four MLPs, trained on persons 2 to 16 and tested on person 1.

Participant	1	2	3	4	5	6	7	8	9	10	11	12	13	14	15	16	Acc.
MLP1-30	81%	50%	69%	55%	76%	59%	56%	68%	79%	68%	88%	72%	95%	72%	87%	79%	72.1%
MLP2-30	83%	51%	72%	60%	79%	62%	58%	72%	85%	74%	89%	73%	97%	75%	91%	83%	75.3%
MLP1-50	83%	49%	69%	58%	79%	62%	57%	68%	84%	70%	89%	75%	90%	74%	90%	80%	74.0%
MLP2-50	86%	52%	74%	63%	83%	65%	60%	72%	89%	74%	90%	75%	98%	75%	93%	82%	77.0%

**Table 8:** Every participant performed each hand gesture ten times. An entry names the number of correctly recognised samples.

	P1	P2	P3	P4	P5	P6	P7	P8	P9	P10
grab	10	6	3	5	5	8	6	7	5	4
release	7	6	9	8	9	8	8	9	9	7
zoom in	10	10	10	10	10	10	10	10	10	10
zoom out	9	10	7	10	9	9	10	8	9	9

**Table 9:** Test results of three investigated CNN models on a total of four different datasets in terms of classification error.

	W150	W100 + 50	ROTARM	REHAP
CNN-2DP	36.1%	41.8%	62.0%	16.1%
CNN-PPC	17.5%	27.4%	55.6%	16.5%
CNN-NPC	39.5%	40.2%	73.3%	25.2%

**Table 10:** The five best results from our Convolutional Neural Network (CNN) parameter grid search in terms of classification error (CE).

Result Rank	$k_{i}^{1}$	$k_{j}^{2}$	$k_{s}^{1}$	$k_{s}^{2}$	$k_{2}^{\mathrm{mp}}$	CE
1	20	30	3	6	1	5.557
2	20	20	3	4	1	5.957
3	20	25	3	6	1	5.971
4	20	35	3	6	1	5.971
5	20	35	3	7	1	5.985

**Table 11:** The best 18 results of our long short-term memory (LSTM) parameter grid search. We conducted a total of 108 experiments, varying the network topology and training parameters.

B	2	5	10	10	5	2	10	5	5	10	10	5	2	5	2	5	5	2
M	1	1	4	3	2	1	3	2	4	1	2	1	4	4	2	4	2	1
C	512	256	128	512	128	256	256	512	128	128	128	512	128	512	512	256	128	128
I	10^4^	10^4^	5 × 10^3^	5 × 10^3^	5 × 10^3^	10^4^	5 × 10^3^	10^4^	10^4^	5 × 10^3^	10^4^	10^4^	10^4^	5 × 10^3^	10^4^	10^4^	10^4^	10^4^
ξ	100	96.7	100	98.3	100	95	96.7	96.7	100	97.5	99.2	100	100	99.2	100	100	95.8	96.7

**Table 12:** Summary of the performances of the methods we investigated in our course of research.

Method	Data Samples (Train/Test)	Training Performance	Test Performance
MLP	100,000/100,000	93.7%	98.7%
SVM	160,000/160,000	N/A	99.8%
Two-stage MLP	160,000/160,000/160,000	97%	77%
CNN	266,000/114,000	94.5%	98.5%
LSTM	480/120	100%	100%

**Table 13:** Selected INTUI questionnaire scores from our human–machine interaction experiment.

	Mean	Standard Deviation
Effortlessness	3.98	0.93
Gut feeling	2.65	1.15
Verbalisation	6.967	0.01
Magic experience	4.85	0.79
Intuition	3.2	1.9

## Abbreviations

The following abbreviations are used in this manuscript:

ToF	time-of-flight
ESF	ensemble of shape functions
PFH	point feature histogram
VFH	viewpoint feature histogram
PCL	point cloud library
MLP	multilayer perceptron
SVM	support vector machine
CNN	convolutional neural network
LSTM	long short-term memory
ML	machine learning
LCT	lane change test
PT	primary task
ST	secondary task
